# Localized Populations of CD8^low/−^ MHC Class I Tetramer^+^ SIV-Specific T Cells in Lymphoid Follicles and Genital Epithelium

**DOI:** 10.1371/journal.pone.0004131

**Published:** 2009-01-05

**Authors:** Jung Joo Hong, Matthew R. Reynolds, Teresa L. Mattila, Aaron Hage, David I. Watkins, Christopher J. Miller, Pamela J. Skinner

**Affiliations:** 1 Department of Veterinary and Biomedical Sciences, University of Minnesota, Saint Paul, Minnesota, United States of America; 2 Wisconsin National Primate Research Center, University of Wisconsin Madison, Madison, Wisconsin, United States of America; 3 University of California Davis, Davis, California, United States of America; New York University School of Medicine, United States of America

## Abstract

CD8 T cells play an important role in controlling viral infections. We investigated the in situ localization of simian immunodeficiency virus (SIV)-specific T cells in lymph and genital tissues from SIV-infected macaques using MHC-class I tetramers. The majority of tetramer-binding cells localized in T cell zones and were CD8^+^. Curiously, small subpopulations of tetramer-binding cells that had little to no surface CD8 were detected in situ both early and late post-infection, and in both vaginally and rectally inoculated macaques. These tetramer^+^CD8^low/−^ cells were more often localized in apparent B cell follicles relative to T cell zones and more often found near or within the genital epithelium than the submucosa. Cells analyzed by flow cytometry showed similar populations of cells. Further immunohistological characterization revealed small populations of tetramer^+^CD20^−^ cells inside B cell follicles and that tetramer^+^ cells did not stain with γδ-TCR nor CD4 antibodies. Negative control tetramer staining indicated that tetramer^+^CD8^low/−^ cells were not likely NK cells non-specifically binding to MHC tetramers. These findings have important implications for SIV-specific and other antigen-specific T cell function in these specific tissue locations, and suggest a model in which antigen-specific CD8+ T cells down modulate CD8 upon entering B cell follicles or the epithelial layer of tissues, or alternatively a model in which only antigen-specific CD8 T cells that down-modulate CD8 can enter B cell follicles or the epithelium.

## Introduction

Rhesus macaques infected with simian immunodeficiency virus (SIV) have been extensively and successfully used as an animal model to help understand the immunopathogenesis of human immunodeficiency virus (HIV) [1], [2]. Many studies have provided strong evidence for the importance of virus-specific CD8^+^ T lymphocytes in controlling viral replication in this animal model. For example, the emergence of CD8^+^ cytotoxic T lymphocytes coincides with reduced viral loads during acute infection [3], [4]. Furthermore, depletion of circulating CD8^+^ lymphocytes during SIV-infected macaques leads to an increase in viremia [5], [6]. For these reasons an effective HIV/AIDS vaccine is thought to require the induction of virus-specific CD8^+^ T lymphocytes [7], [8]. However, we lack a complete understanding of in vivo localization and abundance of virus-specific CD8 T cell responses during SIV infection at the portal of virus entry and in lymphoid tissues. Understanding SIV-specific CD8 T cell localization will help us understand the role of virus-specific CD8 T cells in controlling viral replication in vivo.

Antigen-specific T cells can be visualized in situ by staining tissue sections with MHC class I tetramers [9], [10]. In previous studies, we used in situ tetramer staining to characterize antigen-specific T cells in tissues from mice [11], primates [12]–[14], and humans [15]. In situ tetramer staining allows researchers to determine the localization of antigen-specific T cells in specific tissue compartments, to determine the relationship of antigen-specific T cells to other cells, and to correlate the phenotype of antigen-specific T cells to specific tissue locations.

In our previous studies, we investigated the in situ localization of SIV-specific T cells in tissues from rhesus macaques infected with SIV using MHC-tetramers and found that most MHC-tetramer stained cells were CD8^+^ and localized with other CD8^+^ T cells in lymphoid and genital tissues [12]–[14], [16]. During the course of these studies, we identified subpopulations of SIV-specific T cells that appear to have down-modulated surface expression of CD8 molecules in B cell follicles and in the vaginal and cervical epithelium. We present these findings here and discuss the importance of these findings to HIV and SIV infections.

## Results

### Identification of unique subpopulations of tetramer^+^CD8^low/−^ cells in situ

**Figure 1:**
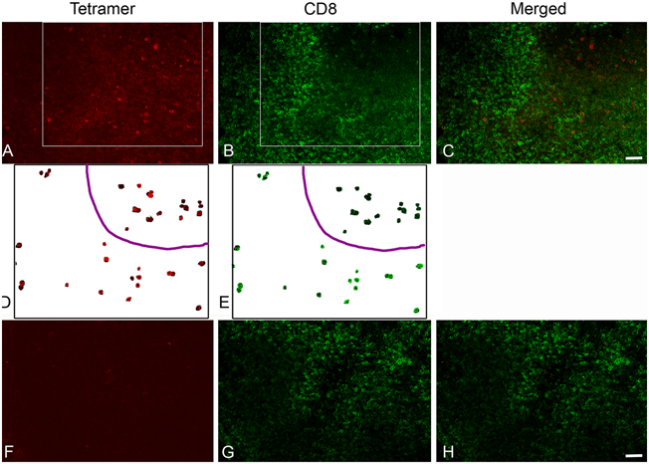
Localization of SIV-specific CD8^low/−^ T cells in lymph nodes. In each set of panels, the left panels show tetramer staining (red), the middle panels show CD8 staining (green), and the right panels merged images of the red and green stain (A) and (D) show Mamu-A*01 Tat SL8 staining and (F) shows staining from the negative control Mamu-A*01 FLP tetramer. Panels (D) and (E) are derived from (A) and (B), but show only the tetramer stained in order to more easily note the lack of CD8 staining on the cells in the cluster of cells in the upper right-hand quadrant of the image delineated by the purple line. These are representative images from axillary lymph nodes from SIV-infected rhesus macaque #27357 at 20 days post-infection. All images are confocal Z-scans collected using a 20× objective. Scale Bar = 50 microns.

**Figure 2:**
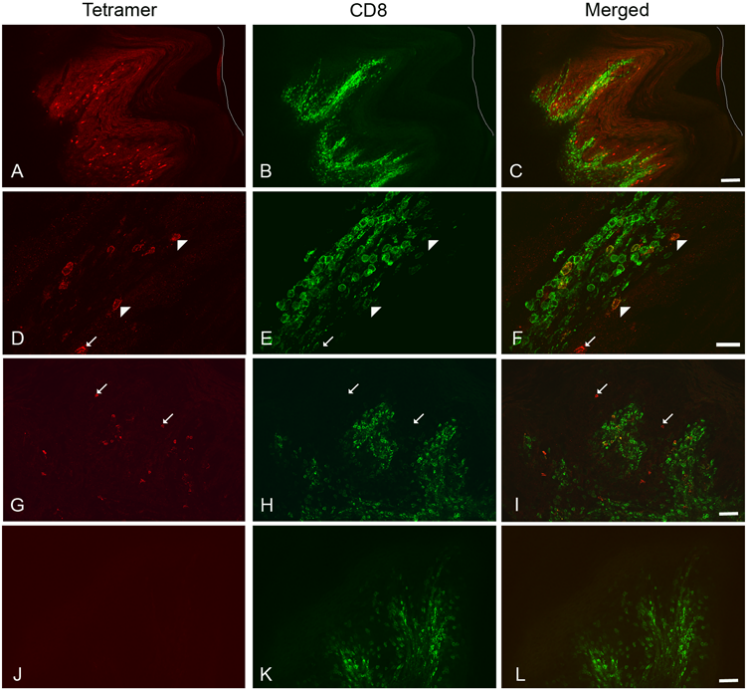
Localization of SIV-specific CD8^low/−^ T cells in the genital tract. In each set of panels, the left panels show tetramer staining (red), the middle panels show CD8 antibody stain (green), and the right panels are merged images of the left and middle images. Mamu-A*01 Gag CM9 staining is shown in (A, D and G) and negative control Mamu-A*01 FLP is shown in (J). All panels are representative images of vagina from animal #27338 at 28 day post-infection, with panels (D) to (F) showing a higher magnification of the area indicated by the white box in (C). White arrow heads in (F) indicate tetramer^+^ CD8^low^ T cells. White arrows indicate tetramer^+^CD8^low/−^ cells. The outer edge of the genital tract epithelium is indicated with a white line. All images are confocal Z-scans collected using a 10× (A to C), 20× (G to L) and 60× objective (D to F). Bars: (A to C) 100 microns, (G to L) 50 microns and, (D to F) 20 microns.

**Figure 3:**
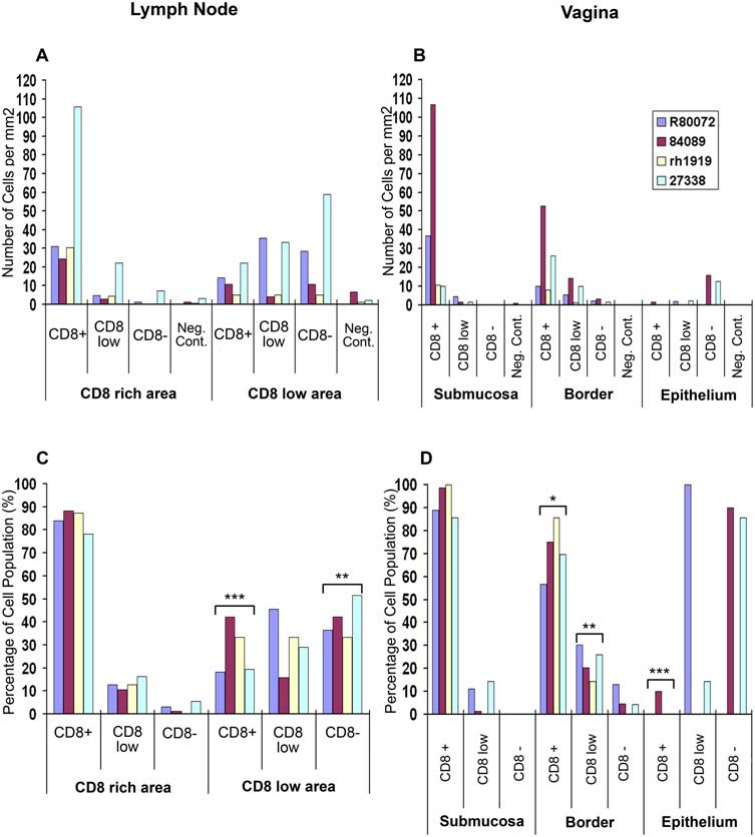
Concentration and localization of SIV-specific CD8 T cells in lymph nodes and vagina. This figure shows the concentration (cells/mm^2^) of tetramer^+^ cells that were CD8^+^, CD8^low^, and CD8^−^ found in CD8 rich and in CD8 low/negative regions of lymph nodes [30], and within the submucosa, border of the submucosa, and within the epithelium of vagina sections (B). (C) and (D) show the percentage of tetramer^+^ cells that are CD8^+^, CD8^low^ and CD8^−^ within each tissue area. All lymph nodes were axillary with the exception of sections from animal #R80072 in which axillary and mesenteric lymph nodes were stained together. Results of t-tests comparing the percentages of each cell type (CD8^+^, CD8^low^ and CD8^−^) found in CD8 rich areas to CD8 low/negative areas of lymph nodes (C), and comparing the percentages of each cell type in the submucosa, to the percentage of cells found in the border of the submucosa and in the submucosa compared to the epithelium in the vagina (D) that showed p-values of <0.001 are indicated with ***, <0.01 with **, and <0.05 with *.

We investigated the localization of SIV-specific T cells stained with MHC-class I tetramers in lymph nodes, spleen, vagina and cervix tissues from SIV-infected Mamu-A*01 rhesus macaques. In each tissue in which tetramer-binding cells were found most of these cells were also CD8^+^ and localized in T cell zones [12], [16] (Figures 1, 2 and 3). However, we also observed small, localized subpopulations of tetramer-binding cells that were CD8^low/−^ (Table 1, Figure 1, Figure 2, and Figure 3). Tetramer^+^CD8^low/−^ cells tended to be clustered together and showed a distinct localization relative to most CD8^+^ T cells. In lymph nodes and spleen tissues, tetramer^+^ CD8^low/−^ cells were frequently found in B cell follicle-like areas–spherical areas near the cortex that showed little to no staining with CD8 antibodies (Figure 1A–E). In the vagina and cervix, although most tetramer staining cells were CD8^+^ and located in the submucosa, small subpopulations of tetramer^+^CD8^low/−^ cells were also detected. These cells typically localized in areas with few CD8^+^ T cells in the epithelium or near the border of submucosa (Figure 2). Some animals showed relatively large numbers of tetramer^+^CD8^low/−^ cells in the epithelium, whereas others showed just a few tetramer^+^CD8^low/−^ cells located near epithelium and submucosa junction (data not shown). Both Tat_28–35_ SL8 (Tat SL8) and Gag_181–189_CM9 (Gag CM9) tetramers stained a subpopulation of CD8^low/−^ cells in situ, indicating that this subpopulation of cells was not specific for one particular type of antigen-specific T cell (data not shown).

**Table 1:** Presence of tetramer+CD8^low/−^ cells in tissues from SIV-infected rhesus macaques.

Animal #	Days post infection	Inoculation site	Vagina/Cervix	Lymph Nodes	Spleen
84089	21 dpi	rectum	**Yes**	**Yes**	N/A
27028	21 dpi	vagina	**Yes**	**Yes**	N/A
27357	21 dpi	vagina	**Yes**	**Yes**	N/A
R80072	21 dpi	rectum	**Yes**	**Yes**	**Yes**
27572	27 dpi	vagina	N/A	**Yes**	N/A
24225	28 dpi	vagina	**Yes**	**Yes**	N/A
27388	28 dpi	vagina	**Yes**	**Yes**	N/A
rh1919	28 dpi	rectum	**Yes**	**Yes**	**Yes**
80025	98 dpi	rectum	N/A	**Yes**	N/A
87108	105 dpi	rectum	**Yes**	**Yes**	N/A
R96111	396 dpi	rectum	**Yes**	N/A	N/A

N/A = not available. We could not distinguish the genital epithelium from lamina propria in sections from animals 27572 and 80025.

### Quantification of unique subpopulations of tetramer^+^CD8^low/−^ cells in situ

To further illustrate the localization and abundance of tetramer^+^ CD8^low/−^ cells, we quantified cells in different locations within lymph node and vagina tissues in four animals (R80072, 84089, rh1919, and 27338). In lymph node and vagina tissue sections stained with Gag CM9 tetramers and anti-CD8 antibodies, we scored tetramer^+^ cells as being CD8^+^, CD8^low^, or CD8^−^. Tetramer^+^CD8^low^ cells were defined as cells that showed little CD8 staining in the membrane that was just above background levels, and tetramer^+^CD8^−^ cells were defined as cells that showed no CD8 staining in the membrane above background levels. The location of cells within tissues was also scored. Cells in the lymph nodes were scored as being either in a CD8 T cell rich region, or within a region morphologically resembling a B cell follicle – a spherical region with few CD8 T cells near the cortex. In the vagina, cells were scored as being in the submucosa, near the border of the submucosa and epithelium or within the epithelium. The results showed that in the lymph node most cells were tetramer^+^CD8^+^ and these cells were most highly concentrated in the T cell rich areas; whereas tetramer^+^CD8^−^ cells were much fewer in number and more highly concentrated in B cell follicle-like areas (Figure 3A). In the vagina, most cells were tetramer^+^CD8^+^ cells and these cells were most highly concentrated in the submucosa (Figure 3B). Tetramer^+^CD8^low^ cells were more highly concentrated in the border of the submucosa and tetramer^+^CD8^−^ cells were more highly concentrated in the epithelium (Figure 3B). Negative control staining was done in lymph nodes with tetramers loaded with an irrelevant peptide for animals r80072, 84089, and rh1919, and with no tetramer for animal 27338. We have previously found that negative control staining with no tetramer and tetramers loaded with an irrelevant peptide show similar results. For vagina sections, negative control staining was done with tetramers loaded with an irrelevant peptide for all four animals. In lymph nodes from each animal the concentration of positively stained CD8^−^ cells in CD8 negative regions was higher in MamuA*01/Gag CM9 stained sections than in the negative control stained sections, with concentrations of Gag CM9 cells showing a 1.4 to 28.4 fold increase over negative control staining (Figure 3A). In the vagina, when present, concentrations of Gag CM9-binding cells were similarly substantially greater than negative control staining for each animal (Figure 3B).

Figure 3C and D shows the percentage of tetramer^+^CD8^+^, tetramer^+^CD8^low^, and tetramer^+^CD8^−^ within each tissue region. In the lymph nodes, the percentage of tetramer^+^CD8^+^ in CD8 rich regions of the lymph node was significantly greater than the percentage of tetramer^+^CD8^+^ cells in CD8 low B cell follicle-like regions (Figure 3C). The percentage of tetramer^+^CD8^low^ cells in the lymph nodes was not significantly different in the two tissue compartments, while the percentage of tetramer^+^CD8^−^ cells was significantly greater within the B cell follicle-like areas compared to the CD8 T cell-rich regions of the lymph nodes (Figure 3C). In the vagina, the percentage of tetramer^+^CD8^+^ cells within the submucosa was significantly greater than the percentage of tetramer^+^CD8^+^ cells near the border of the submucosa and epithelium or in the epithelium (Figure 3D). Also, the percentage of tetramer^+^CD8^low^ cells near the border of the submucosa was significantly greater than the percentage of cells that were tetramer^+^CD8^low^ in the submucosa (Figure 3D).

In addition, to better quantify the number of times clusters of tetramer^+^CD8^low/−^ cells were present within B cell follicles in lymph nodes as determined by morphology (a spherical area with few CD8 T cells near the cortex), using a confocal microscope we collected a z-scan image through the middle of each apparent follicle in stained sections and scored the number of tetramer^+^CD8^low/−^ cells found within. The results showed that 75% (18 of 24) B cell follicle-like areas identified showed clusters of 3 to 13 tetramer^+^CD8^low/−^ cells within. In contrast, similarly examined negative control sections did not show any clusters of tetramer^+^CD8^low/−^ cells in 18 follicle-like areas examined. Representative images are shown in Figure 1.

**Figure 4:**
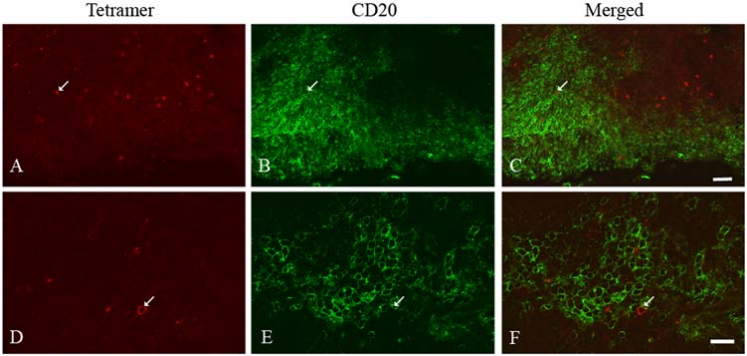
Localization of SIV-specific T cells in B cell follicle. In each set of panels, the left images (A and D) show Mamu-A*01 Gag CM9 tetramer staining (red), the middle images (B and E) show CD20 antibody staining (green), and right images (C and F) are merged image of the left and middle images. The bottom panels shows a higher magnification image taken from within the large B cell follicle shown in the top panels. The white arrows point to a tetramer+CD20^−^ cell within the B cell follicle. Representative images are from an axillary lymph node from animal #24225 at 28 day post-infection. Confocal images were collected using a 20× (A to C) and 60× objective (D to F). Bars: (C) 50 microns, (F) 20 microns.

When we stained these sections with MamuA*01/Gag CM9 tetramers and CD20 antibodies (B cell marker) to determine whether tetramer^+^ cells localized to B cell follicles, we found small populations of tetramer^+^ cells in B cell follicles (Figure 4). These data taken together with the data presented in figures 1 and 3 suggest that the tetramer^+^CD8^low/−^ cells detected in lymphoid tissues were localized to B cell follicles.

### Tetramer staining of disaggregated cells from lymph and genital tissues

**Figure 5:**
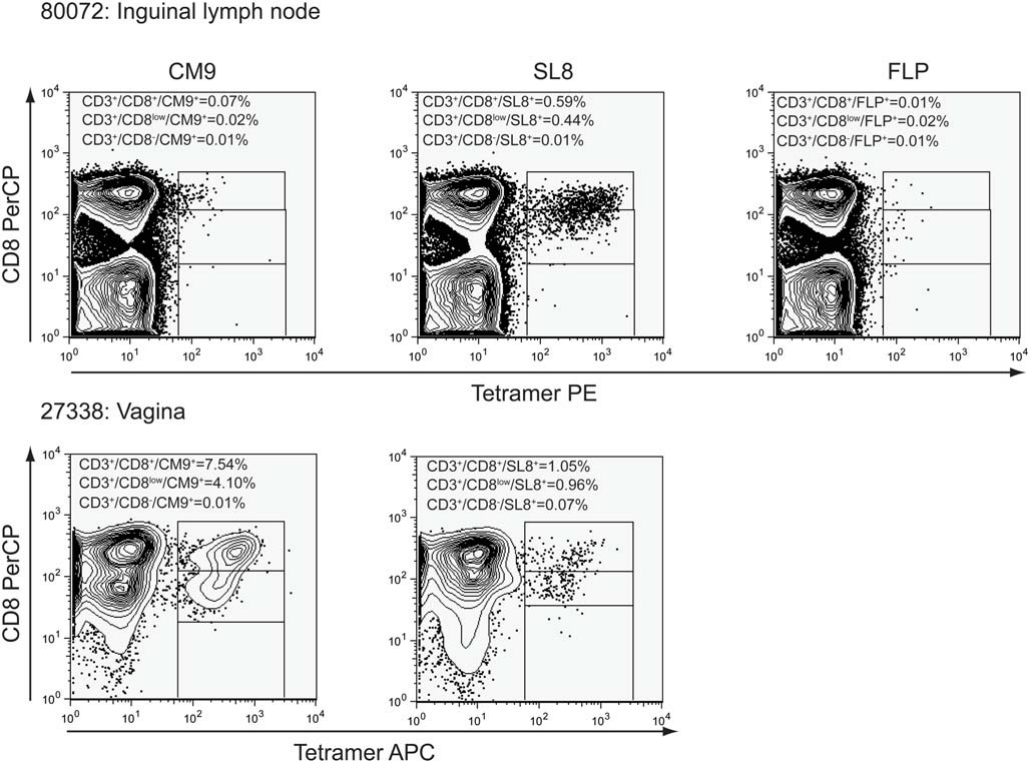
Tetramer^+^CD3^+^CD8^low/−^ stained cells from disaggregated lymph nodes and vagina analyzed by flow cytometry. Disaggregated cells from SIV-infected macaques were stained with Mamu-A*01 Gag CM9, Mamu-A*01 Tat SL8, and negative control Mamu-A*01 FLP tetramers; counterstained with CD3 and CD8 antibodies and analyzed by flow cytometry. Populations of SIV-specific tetramer^+^CD3^+^CD8^low/−^ lymphocytes were not detected above negative control staining. However, populations of tetramer^+^CD3^+^CD8^low^ cells were detected above background levels. Representative data from inguinal lymph node from animal #R80072, and vaginal submucosa from #27338 is shown. Note negative control staining FLP was not done with the vaginal submucosal cells.

**Figure 6:**
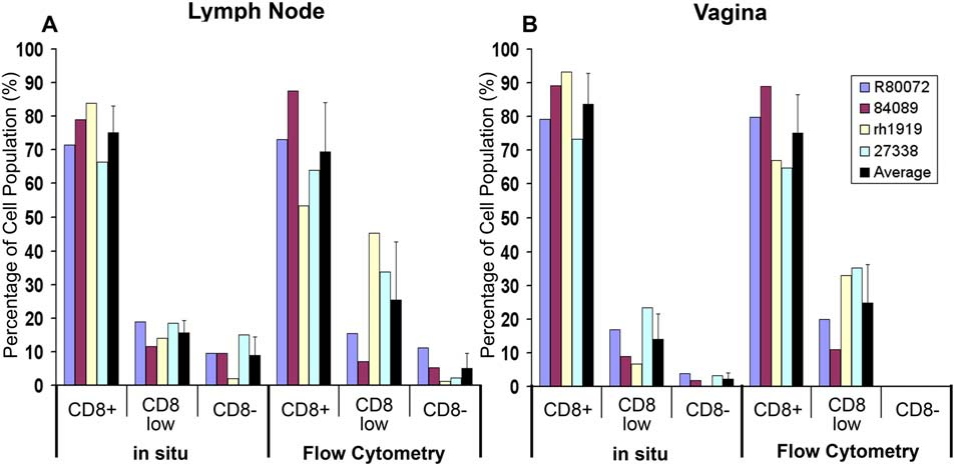
Comparison of populations observed via in situ tetramer staining to flow cytometry. The percentage of tetramer^+^ cells that were CD8^+^, CD8^low^, and CD8^−^ within lymph nodes (A) and vagina submucosa tissues (B) using in situ tetramer staining and flow cytometry. For in situ tetramer staining, all lymph nodes were axillary with the exception of animal #R80072 in which axillary and mesenteric lymph nodes were stained together. For flow cytometry, all lymph nodes were axillary except #27338 which was inguinal.

We also examined whether similar populations of tetramer^+^CD8^low/−^ cells were detected in disaggregated cells using flow cytometry. The percentage of tetramer^+^CD8^−^ cells was equal to or less than 0.08% of the CD3^+^ lymphocytes isolated from lymph nodes and were not above the levels of background staining with negative control tetramers loaded with an irrelevant peptide (Figure 5), nor were tetramer^+^CD8^−^ populations without gating on CD3 (data not shown). However, we consistently saw populations of tetramer^+^CD3^+^CD8^low^ cells above levels of negative control tetramer staining (Figure 5). To better compare results from in situ tetramer staining with flow cytometry, we calculated the percentage of tetramer^+^ cells that were CD8^+^, CD8^low^, and CD8^−^ within the lymph nodes and vagina tissues using in situ tetramer staining and flow cytometry. Because the vaginal tissues processed for flow cytometry had the epithelium removed and thus only contained cells from the submucosa, for this comparison we only included cells in the submucosa or border of the submucosa for our in situ tetramer staining calculations. The results showed no statistically significant differences in the populations tetramer^+^CD8^+^, tetramer^+^CD8^low^, and tetramer^+^CD8^−^ cells calculated via in situ tetramer staining compared to flow cytometry (Figure 6), indicating that the populations detected via in situ tetramer staining are also detected using flow cytometry.

### The presence of tetramer^+^CD8^low/−^ cells in situ was not affected by route of inoculation or time post-infection

In this study we examined tissues from animals that were vaginally inoculated and animals that were rectally inoculated with SIVmac239. We also examined tissues from animals that were sacrificed at relatively early and late post-infection. Tetramer^+^ CD8^low/−^ cells were identified in both vaginally and rectally inoculated rhesus macaques early after infection (Table 1), indicating that the route of inoculation does not affect the formation of this novel subpopulation of cells. Tetramer^+^ CD8^low/−^ cells were also present in lymph nodes and genital tract early and late post-infection (Table 1) indicating that time post-infection did not affect this novel population of cells.

### Tetramer^+^ CD8^low/−^ cells are not likely NK cells, B cells, CD4^+^T cells or γδ T cells

**Figure 7:**
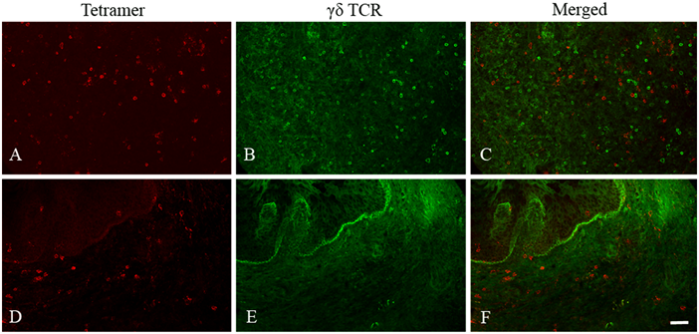
Localization of SIV-specific T cells and γδ TCR+ cells in lymph node and vagina. In each set of panels, the left images (A and D) are Mamu-A*01 Gag CM9 tetramer stain (red), the middle image (B and E) are γδ TCR antibody stain (green), and right images (C and F) are merged images of the left and middle images. Panels A to F are representative images from animal #27572 that show staining in inguinal lymph node and vagina at 27 day post-infection. Images are all confocal Z-scans collected using a 20× objective. Bar = 50 microns.

**Figure 8:**
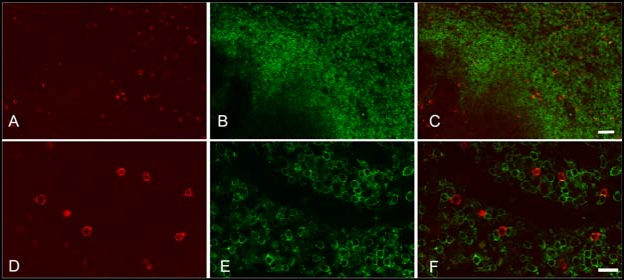
Localization of SIV-specific T cells and CD4^+^ T cells in lymph node. In each set of panels, the left images (A and D) show Mamu-A*01 Gag181–189 CM9 tetramer staining (red), the middle images (B and E) show CD4 antibody staining (green), and right images (C and F) are merged images of the left and middle images. Images are from a genital lymph node of animal #28850. Confocal images collected using a 20× objective (A to C) and 60× objective (D to F). Bars: (A to C) 50 microns, (D to F) 20 microns.

To determine whether tetramer^+^CD8^low/−^ cells were possibly NK cells non-specifically binding MHC class I tetramers, tissue sections were stained with negative control tetramers loaded with a peptide derived from the hepatitis B virus core. These stained sections did not show populations of tetramer^+^CD8^low/−^ cells as were detected with the Gag CM9 and Tat SL8 tetramers (Figure 1f–h, Figure 2j–l, Figure 3). These data indicate that the tetramer^+^CD8^low/−^ cells were not NK cells non-specifically binding tetramers. To determine whether the tetramer^+^CD8^low/−^ cells were possibly B cells, γδ TCR T cells, or CD4+ T cells, we stained tissue sections with anti-human CD20 (Figure 3), γδ TCR (Figure 7), and CD4 (Figure 8) antibodies and tetramers. Tetramer^+^ cells in lymph nodes and the genital tract were not counterstained with any of these antibodies indicating that tetramer^+^CD8^low/−^ cells detected in situ are none of these cell types.

## Discussion

CD8 molecules on T cells are associated with signal transduction through the interaction between peptide MHC class I complex and T cell receptor (TCR), leading to T cell activation [17]. Once activated, CD8^+^ T cells can kill cells that express the specific MHC-class I molecules and peptide recognized by T cell receptors [18]. We have previously reported that MHC class I tetramers bound to viral peptides can be used to detect virus-specific T cells in situ in tissues from rhesus macaques infected with SIV and lymph nodes from individuals infected with HIV [12]–[15]. We found that the majority of tetramer staining cells in situ expressed CD8 molecules and localized to T cells zones. Here, we describe the observation of small localized subpopulations of cells that stain with tetramers but are CD8^low/−^ in situ. These cells were observed in lymph node, spleen, vagina, and cervix tissues. In lymph node and spleen tissues, these cells often appeared to be in or next to B cell follicles. In genital tissues, tetramer^+^CD8^low/−^ cells were primarily localized within the epithelium or near the border of the epithelium and submucosa.

Tetramer^+^CD8^low/−^ subpopulations of cells were detected in lymph node, spleen, vagina and cervix tissues at both early and late time points post-infection (Table 1). Tetramer^+^CD8^low/−^ cells were also detected in rhesus macaques that were vaginally or rectally inoculated. These findings suggest that the time post-infection and route of inoculation did not affect the presence and histological pattern of tetramer-stained cells showing little to no detectible CD8 molecules on their cell surfaces.

In order to confirm the specificity of tetramer staining, we used MHC class I Mamu A*01 tetramers loaded with the irrelevant peptide (FLPSDYFPSV) from viral hepatitis B virus core protein as a negative control. This allowed us to distinguish between specific and nonspecific staining in our experiments. Sections stained with the negative control tetramers did not show clusters of tetramer^+^CD8^low/−^ cells as was found in sections stained with virus-specific tetramers. These results indicate that the tetramer^+^CD8^low/−^ cells detected in situ in our experiments were not NK cells that non-specifically bound to the MHC class I molecules in the MHC class I tetramers.

Although it is unlikely that the tetramer^+^ CD8^low/−^ cells detected in our experiments were γδ T cells because these cells are not MHC-class I restricted [19], and most simian γδ T cells express CD8 molecules [20], we nonetheless set out to eliminate this possibility. It is 1) conceivable that some γδ T cells might recognize a peptide presented by classical class I MHC proteins or MHC class I like proteins that is generally characteristic for αβ T cells [21] and 2) known that most γδ T cells are CD8^low/−^ negative in humans [22]. We found that tetramer stained cells were not co-labeled with γδ TCR-antibodies in lymph nodes or the genital tract, indicating that the tetramer^+^ CD8^low/−^ cells were not γδ T cells.

Tetramer^+^ CD8^low/−^ cells in secondary lymphoid organs often appeared to be located near or within the B cell follicles. Although most tetramer stained cells were CD8^+^ and localized in T cell zones, the observed tetramer^+^ CD8^low/−^ cells tended to localize in non-T cells zones that resembled B cell follicles in lymph node and spleen tissues. Sections stained with tetramers and CD20 antibodies showed that there were indeed small populations of tetramer^+^ cells located within B cell follicles in lymph tissues. These results taken together suggest that tetramer^+^ CD8^low/−^ populations of cells in secondary lymph tissues are often localized next to or within B cell follicles.

It is not clear what mediates localized populations of tetramer^+^ CD8^low/−^ cells, however, one possible mechanism mediating decreased levels of CD8 on tetramer stained cells in specific tissue locations is the relative abundance of interleukin 7 (IL-7) in specific tissue locations. Recent findings showed that IL-7 modulates CD8 levels on T cells, and high IL-7 levels lead to increased levels of CD8 expression [23]. It has also recently been shown that in secondary lymphoid organs IL-7 is expressed predominantly by fibroblastic reticular cells in the T cell zone [24]. Thus, decreased CD8 levels detected on tetramer stained localized to non-T cell zones, may be a consequence of decreased IL-7 levels. In addition, TCR signaling leads to lower levels of surface CD8 molecules [23], [25], [26]. Furthermore, B cell follicles contain many antigen presenting cells that antigen-specific T cells within B cell follicles come into contact with. It is also therefore possible that tetramer^+^CD8^low/−^ cells detected in situ were undergoing TCR engagement and as a consequence showed decreased CD8 levels. Thus, local IL-7 levels and TCR engagement are both possible mechanisms that may contribute to the observed tetramer^+^CD8^low/−^ cells.

It is not clear why there exist localized populations of tetramer^+^ CD8^low/−^ cells. Because CD8 molecules are associated with TCR signaling and T cell function, one can reason that tetramer^+^ CD8^low/−^ cells have altered function relative to tetramer^+^ CD8^high^cells. Quigley et al., recently showed that a subset of CD8^+^ T cells express the chemokine receptor CXCR5 and localize to B cell follicles in human tonsils [27]. The CXCR5^+^CD8^+^ T cells showed an early effector non-cytolytic memory phenotype. When stimulated in vitro these cells expressed the B cell interacting molecules CD70, OX40 and ICOS, and these cells also supported the survival of B cells in culture suggesting a role of memory CD8 T cells in B cell follicles. Also, recent studies by Khanna et al, used in situ tetramer staining to determine the kinetics and localization of antigen-specific CD8 T cells in spleens of *Listeria monocytogenes*-infected mice [28]. They showed that 60% of the memory tetramer^+^ CD8^+^ T cells localized to B cell follicles in the spleen, and 48-hours post-restimulation the cells migrated out of the B cell follicle and into the T cell zone and red pulp. These studies suggest that the SIV-specific tetramer stained cells we detect in B cell follicles from SIV-infected rhesus macaques may be SIV-specific memory T cells.

To our knowledge, this is the first description of localized subpopulations of tetramer^+^ CD8^low/−^ cells identified in tissues. These findings have important implications for understanding CD8 T cell function, and for understanding the role of CD8 T cells in the immunopathogenesis of HIV and other infectious diseases involving infections that take place within lymphoid follicles and within the genital epithelium. These findings suggest a model in which antigen-specific CD8^+^ T cells down-modulate CD8 upon entering B cell follicles or upon entering the epithelial layer of tissues in macaques or alternatively, a model in which only cells that have down-modulated CD8 can enter these tissue locations.

## Materials and Methods

### Animals

Adult female rhesus macaques (*Macaca mulatta*) were maintained at the California National Primate Research Center and the Wisconsin National Primate Research Center in accordance with the regulations of the American Association of Accreditation of Laboratory Animal Care standards. The study was approved by local institutional animal use and care as well as biosafety review boards. We investigated Mamu-A*01 rhesus monkeys that were either intravaginally or intrarectally infected with SIVmac239 and sacrificed either during the acute or chronic stages of infection as previously described [12], [16], [29]. Freshly dissected lymph nodes, spleen, vagina and cervix tissues were shipped on ice in RPMI containing 100 µg/ml heparin over night from either the National Primate Research Center in either California or in Wisconsin to the Skinner lab in Minnesota.

### In situ tetramer and immunohistochemical staining

Fresh lymph nodes, spleen, vaginal and cervical tissues were used throughout these studies. In situ tetramer staining was performed essentially as previously described [11], [12] to detect SIV-specific T cells in tissues from rhesus macaques. We purchased biotinylated Mamu-A*01 molecules loaded with SIV gag (CTPYDINQM) peptides (Immunomics), and loaded with SIV tat (STPESANL) and irrelevant (FLPSDYFPSV) peptides (National Institute of Allergy and Infectious Diseases tetramer facility). Tetramers were generated by adding six aliquots of FITC-labeled ExtraAvidin (Sigma) to biotinylated Mamu-A*01/β2m/peptide monomers over the course of 8 h to a final molar ratio of 4.5∶1. For in situ tetramer staining, fresh tissues were cut into approximately 0.5-cm pieces and embedded in 4% low-melt agarose. Tissue blocks were placed in a vibratome bath containing 0 to 4°C PBS with 100 ug/ml heparin (PBS-H), and 200 um thick sections were generated. Sections were incubated at 4°C overnight with tetramers (0.5 ug/ml) and either mouse anti-human CD8 alpha chain (Dako clone DK25), CD20 (Novocastra Laboratories Ltd), CD4 (Lab Vision clone 1F6), or γδ TCR (Biosciences) antibodies diluted 1∶200 in PBS-H with 2% normal goat serum (NGS). Sections were then washed with chilled PBS-H and fixed with 4% paraformaldehyde for 2 h at room temperature. Sections were again washed with PBS-H, incubated with rabbit anti-FITC antibodies (BioDesign) diluted 1∶10,000 in PBS-H with 2% NGS, and incubated at 4°C on a rocking platform overnight. Sections were washed three times with PBS-H for at least 20 min and then incubated with Cy3-conjugated goat anti-rabbit antibodies diluted 1∶5000 (Jackson ImmunoResearch) and Alexa 488-conjugated goat anti-mouse antibodies diluted 1∶2000 (Molecular Probes), in PBS-H with 2% NGS, for 1 to 3 days. Finally, sections were washed three times for at least 20 min in PBS-H, post-fixed with 4% paraformaldehyde for 1 h, and then mounted on slides with warmed glycerol gelatin (Sigma) containing 4 mg/ml *n*-propyl galate. Stained sections were analyzed using a Bio-Rad 1000 or 1024 confocal microscope. Digital images were collected and analyzed using Confocal Assistant version 4.02 and Adobe Photoshop 7.01.

### Quantitative Image Analysis

For each tissue section stained with tetramers and CD8 antibodies, images were collected with an Olympus FluoView1000 confocal microscope using a 20× objective. At least three fields were collected for each tissue, and if possible more fields (as many as 9) were collected. Using FluoView software, tetramer positive cells were scored in each image as being CD8^+^, CD8^low^ and CD8^−^. Tetramer^+^CD8^low^ cells were defined as showing levels of CD8 staining in the membrane that was just above background levels, and tetramer^+^CD8^−^ cells were defined as cells that showed no CD8 staining in the membrane above background levels. The location of cells within tissues was also noted. Cells in lymph nodes were scored as being either in a CD8+ rich region, or within a spherical region with few CD8 T cells near the cortex of the tissue resembling a B cell follicle. In the vagina, cells were scored as being in the submucosa, near the border of the submucosa and epithelium or within the epithelium. Cells near the border of the submucosa were defined as cells in the submucosa that were within 30 um (approximately three cells) from the border of the submucosa and epithelium. In addition, to quantify the number of times clusters of tetramer^+^CD8^low/−^ cells were present in apparent B cells follicles, we examined lymph node sections for regions with B cell morphology (little CD8 staining, spherical, and near the cortex) and collected a z-scan through the middle of the apparent follicle and scored the number of tetramer^+^CD8^low/−^. Statistical analysis was done using a two-tailed T-test.

### Flow Cytometry of Tetramer stained cells

To determine the proportion of cells that were CD3^+^ tetramer^+^ and CD8^low/−^ cells in populations of lymphocytes isolated from lymph node and vagina tissues we performed tetramer staining as previously described [12]. Briefly, frozen lymphocytes from lymph nodes were thawed and washed twice with RPMI (Cambrex) with 10% fetal bovine serum. Then 5×10^5^–1×10^6^ cells were incubated with Mamu-A*01/Gag CM9, Mamu-A*01/Tat SL8, or Mamu-A*01/FLP tetramers labeled with phycoerythrin for 1 hour at 37°C. The lymphocytes were then incubated with anti-human CD3ε-FITC (SP34; Pharmingen) and anti-CD8α-PerCP (clone SK1; Becton Dickinson) for 40 min at room temperature. Similar staining with Gag CM9 and Tat SL8 tetramers was performed with cells from the vaginal submucosa. Data collection and analysis was performed using a FACSCalibur flow cytometer (Becton Dickinson) and FlowJo software (Treestar Inc.), respectively.
